# The Effectiveness of Virtual Reality on Anxiety and Pain Management in Patients Undergoing Cardiac Procedures: A Systematic Review and Meta-Analysis

**DOI:** 10.7759/cureus.57557

**Published:** 2024-04-03

**Authors:** Zubair S Bashir, Chelsea Misquith, Phinnara Has, Syed M Bukhari

**Affiliations:** 1 Department of Medicine, Warren Alpert Medical School, Brown University, Providence, USA; 2 School of Public Health, Brown University, Providence, USA; 3 Lifespan Biostatistics, Epidemiology and Research Design, Rhode Island Hospital, Brown University, Providence, USA; 4 Vascular Medicine, Cleveland Clinic, Cleveland, USA

**Keywords:** patient education, pain, anxiety, cardiac procedures, virtual reality

## Abstract

Cardiac procedure-related anxiety and pain can adversely affect outcomes and lead to patient dissatisfaction. Virtual reality (VR) offers a promising alternative to traditional therapies for improving patient experience. Our objective is to synthesize evidence and assess the effectiveness of VR in reducing cardiac procedure-related anxiety and pain compared to standard of care. We conducted a comprehensive search across various online databases, including MEDLINE, EMBASE, CINAHL, Web of Sciences, and COCHRANE, to identify relevant randomized controlled trials (RCTs) focusing on VR, cardiac procedures, anxiety, and pain. We utilized a random-effect model to generate effect estimates reported as standardized mean differences (SMD) with a 95% confidence interval. Our review comprised 10 studies with a total of 621 participants (intervention arm: 301, control arm: 320). Overall, among the seven studies evaluating anxiety outcomes, no significant difference in anxiety reduction was observed between the intervention and control groups (standardized mean difference (SMD) -0.62, 95% CI -1.61, 0.37, p=0.22). However, studies using the same anxiety assessment tool demonstrated a significant improvement in the VR arm (SMD -1.01, 95% CI -1.98, -0.04, p=0.04). Conversely, the narrative synthesis of four studies examining pain revealed mixed results. Our findings suggest no significant difference in anxiety reduction between the VR and control groups. Future studies should employ standardized tools for assessing and reporting anxiety and pain to better understand the potential of VR in enhancing patient experience during cardiac procedures.

## Introduction and background

Anxiety is associated with an increased prevalence of cardiovascular disease [1]. This disabling condition manifests with a spectrum of psychosomatic symptoms, some of which closely resemble those related to cardiovascular diseases [2], posing a diagnostic challenge for clinicians and adding to patients' distress. Anxiety has been shown to cause autonomic dysfunction, leading to reduced variability in heart rate, increased blood pressure, and elevated baseline pulse rate [3-5]. These symptoms increase the likelihood of incident cardiovascular disease and also exacerbate underlying heart conditions, resulting in poor outcomes [6-8].

Vogelzangs et al. reported 2.5 to 3.5 times higher odds of coronary heart disease (CHD) among individuals with anxiety symptoms compared to those without [1]. Furthermore, the severity of anxiety symptoms, rather than the duration of anxiety disorder, is strongly associated with CHD [1], emphasizing the importance of effectively controlling anxiety symptoms during cardiac procedures.

A significant percentage, ranging from 32% to 49%, of patients undergoing cardiac procedures experience anxiety symptoms, often attributed to inadequate pain control and insufficient procedural understanding [9]. Effective pain management is essential not only for patient satisfaction but also for overall well-being [10]. Additionally, the psychological distress associated with anxiety and pain can hinder patients' ability to comprehend and retain crucial healthcare information provided before or after the procedure.

To address these challenges, various pharmacological and non-pharmacological techniques have been employed. Among them, virtual reality (VR) emerges as a promising non-pharmacological approach, offering an effective and visually engaging distraction method [11,12]. VR also provides an interactive interface that enhances procedural understanding and has demonstrated efficacy in mitigating anxiety and pain across various medical settings [13-15] including cardio-pulmonary rehabilitation and surgical procedures [16-18]. However, its effectiveness in cardiac procedures has yielded conflicting results [19-21]. Therefore, this systematic review and meta-analysis aim to provide a comprehensive assessment of VR's effectiveness in alleviating anxiety and pain across all types of cardiac procedures.

The preliminary findings of this article were previously presented as a meeting abstract at the 2023 European Society of Cardiology (ESC)Annual Scientific Meeting on August 25, 2023.

## Review

Methods and analysis

This systematic review and meta-analysis adhered to the PRISMA guidelines for reporting [22]. The protocol for the systematic review was prospectively registered with the International Prospective Register of Systematic Reviews (PROSPERO) on 02/11/2023. (Registration number: CRD 42023395395).

Search strategy and participants

A comprehensive search strategy developed by a health sciences librarian was employed to identify relevant articles, which were then imported into Covidence/EndNote20. This strategy incorporated a combination of keywords and controlled vocabulary terms relating to different types of cardiac procedures, pain, anxiety, and virtual reality. The search encompassed several academic research databases, including MEDLINE (via Ovid and PubMed), CINAHL (via EBSCO), Cochrane Central Register of Controlled Trials (CENTRAL), Web of Sciences, and EMBASE (via www.embase.com). Additionally, searches conducted in MEDLINE, CINAHL, and EMBASE utilized validated search filters for randomized controlled trials (RCTs) [23-25]. All databases were searched from inception until the time of final analysis (up to September 30, 2023) to ensure that all the relevant articles meeting our predetermined criteria were included.

Inclusion and exclusion criteria

Our inclusion criteria followed the Participants, Intervention, Comparator, and Outcome (PICO) framework (Table 1). We included adult patients (>18 years) undergoing all types of cardiac procedures and VR technology was part of the procedural care program for managing anxiety and pain. Only RCTs published in the English language were included. We excluded all the clustered control trials, observational studies, case series, case reports, and RCTs published in non-English language.

**Table 1:** PICO framework for eligibility criteria PICO: Participants, Intervention, Comparator, and Outcome; RCTs: Randomized Controlled Trials. VR: Virtual Reality.

Study Selection	We included only RCTs published in the English language from inception till the formal analysis and excluded clustered control trials, observational studies, case series, and case reports.
Participants	Adult patients (>18 years) undergoing all types of cardiac procedures were included.
Intervention	The intervention group utilized various immersive and non-immersive VR technologies to manage anxiety and pain associated with cardiac procedures.
Comparator	The comparator group received standard care for managing anxiety and pain following the guidelines of their respective hospitals for cardiac procedures.
Outcomes	Anxiety and pain levels were evaluated utilizing both standardized and non-standardized commonly utilized questionnaires.

Description of groups

The VR group used various immersive or non-immersive VR technologies to manage anxiety and pain in patients undergoing cardiac procedures. The control group followed the regular anxiety and pain management protocol used by the respective hospitals for patients undergoing cardiac procedures.

Study selection

The full text of all non-duplicate articles was reviewed independently by two reviewers (ZB and SB) based on the inclusion and exclusion criteria. Any conflicts between the reviewers were resolved through discussion. The data extraction strategy was similar to our previous protocols [26,27]. The data for the virtual reality-based anxiety and pain management intervention and control groups was entered into a Microsoft Excel Spreadsheet with designated headings. The information included the total population of both groups and peri-procedural differences in anxiety and pain scores. To ensure consistency, a higher standard deviation (SD) of the outcome measures was recorded in the spreadsheet as the SD of the mean difference.

Outcome measures

Anxiety and pain were the outcomes assessed and the outcome assessment methodology was similar to our previous protocols [26,27]. The outcomes were evaluated by assessing peri-procedural differences in anxiety and pain scores using standardized and non-standardized questionnaires. Articles utilizing non-quantitative assessment methods or demonstrating significant heterogeneity in outcome assessment or reporting were included in the narrative synthesis.

Quality and risk of bias

The Revised Cochrane Risk of Bias tool was utilized to evaluate bias in the included studies [28]. This tool assesses articles across five components: bias arising from the randomization process, bias due to deviations from intended interventions, bias due to missing outcome data, bias in the measurement of the outcome, and bias in the selection of the reported result. Each domain was evaluated and categorized into one of three levels of risk: Low risk of bias, Some concerns, or High risk of bias. Furthermore, the overall risk of bias across all studies was reported.

Data synthesis

The analysis was conducted per protocol assessment. Continuous variables were described using mean and standard deviation (SD). For both anxiety and pain outcomes, pooled results were calculated using standardized mean differences (SMD) with a 95% confidence interval. In cases where heterogeneity was insignificant, effect estimates were computed using a fixed-effect model. Conversely, a Der Simonian and Laird random-effect model was used with significant heterogeneity between studies. A quantitative assessment of heterogeneity was performed using Q statistics [29], with a value exceeding 60% indicating significant heterogeneity. Publication bias was evaluated using Egger’s regression test [30], and a p-value below 0.05 was considered statistically significant. Subgroup and sensitivity analyses were carried out based on various factors, including the type of outcome assessment questionnaire, timing of outcome assessment in relation to the cardiac procedure, and the use of VR for distraction or procedure-related education. Due to the limited number of studies (less than 10) for each outcome, certainty of evidence was not assessed. Data analysis was conducted using Stata SE version 17.0 (College Station, TX, USA) and ReVMan, version 5 (Review Manager, The Cochrane Collaboration, available at www.revman.cochrane.org).

Results

Study Selection

**Figure 1:**
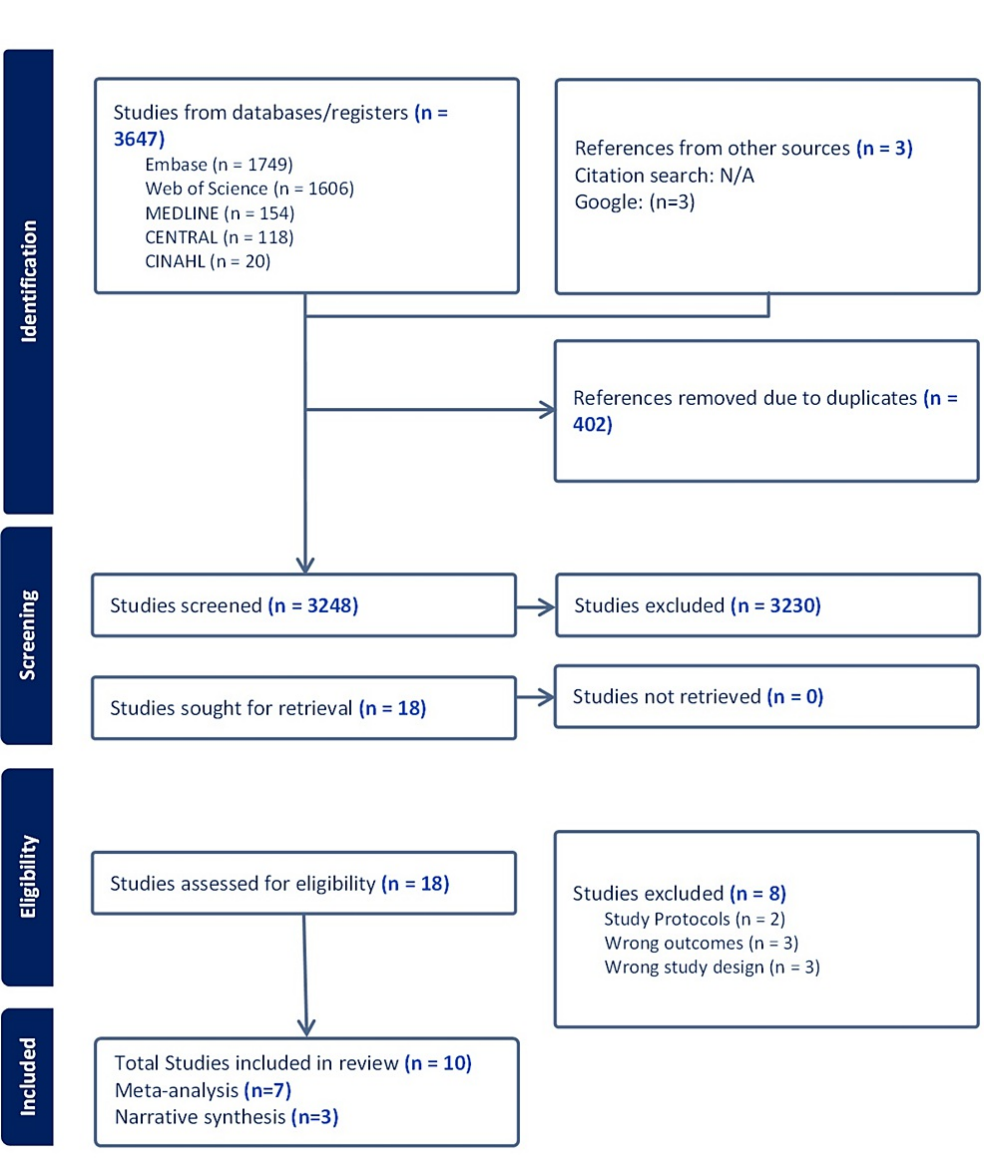
PRISMA flow diagram of study selection PRISMA: Preferred Reporting Items for Systematic Reviews and Meta-Analyses

The study selection process was conducted according to the Preferred Reporting Items for Systematic Reviews and Meta-Analyses (PRISMA) flow diagram shown in Figure 1. Initially, a comprehensive search strategy yielded a total of 3,647 articles. Specifically, Web of Science identified 1,606 articles, EMABSE found 1,749, Medline included 154, Cochrane contained 118, CINAHL contributed 20 studies, and three studies were found through other sources. After removing 402 duplicate articles, the remaining 3,248 articles underwent a screening process. Articles that did not meet the inclusion criteria were filtered out, resulting in a selection of 18 studies. Subsequently, the full text of these articles was reviewed by two independent reviewers (ZB, SB), who assessed them based on the inclusion and exclusion criteria. From this selection process, a total of 10 studies were identified, and out of these, seven studies were included in the meta-analysis [21,31-36], while three studies were included in the narrative synthesis [19,20,37].

Study Characteristics

A summary of the randomized controlled trials (RCTs) included in this review can be found in Table 2. A total of 621 study participants were included across all studies, with the mean age ranging from 43.1±12.0 to 83±6.5 years. All 10 studies assessed anxiety outcomes, while four studies also assessed pain outcomes [19,20,31,37]. Among the 10 studies, five assessed outcomes before the cardiac procedure [21,32,34-36], while the remaining studies used questionnaires to assess outcomes at various time points: before, during, and after the cardiac procedure. Regarding the specific procedures, four studies included participants undergoing coronary artery bypass grafting (CABG) or valve replacement procedures [31,35-37]. One study each focused on atrial fibrillation catheter ablation [19], patent foramen ovale (PFO)/atrial septal defect (ASD) closure [21], and post-cardiac surgery drain removal [20]. The remaining three studies involved participants undergoing coronary angiography or cardiac catheterization procedures [32-34]. In terms of geographical distribution, two studies were conducted in Iran [32,34] and Germany [36,37] each, while one study each was conducted in Belgium [31], France [20], the Netherlands [21], Taiwan [19], the United States [35], and the United Kingdom [33].

**Table 2:** Study characteristics VR: Virtual Reality, HMD: Head Mounted Device, STAI: State and Trait Anxiety Index, VAS: Visual Analogue Score, NRS: Numeric Rating Scale, BHF: British Heart Foundation, CABG: Coronary Artery Bypass Graft, SAVR: Surgical Aortic Valve Repair, TAA: Thoracic Aortic Aneurysm repair, MVR: Mitral Valve Replacement, AVR: Aortic Valve Replacement, PFO: Patent Foramen Ovale, ASD: Atrial Septal Defect, TAVI: Transcatheter Aortic Valve Implantation.

Study Author	Number of participants (Intervention/Control)	Mean Age in years (Intervention /Control)	Cardiac Procedure	Intervention/Control Group Characteristics	Assessment and Outcome
Grab et al., 2023 [36]	65 (31/34)	65.97±8.02/62.94 ±13.94	CABG, SAVR, TAA	Intervention: Education using VR goggles, VR application, VR room with a microphone and allowing free movement in the VR room. Control: Education using standardized pre-printed paper-based models	Pre-surgical Anxiety Questionnaire: Short form STAI (German Version) Second time point was right after patient education and before the cardiac procedure
Pool et al., 2022 [21]	50 (25/25)	44.5±9.9/43.1±12.0	PFO/ASD Closure	Intervention: In addition to oral information from the treating cardiologist and the informative flyer, patients viewed a 5-minute educational VR video. VR headsets were used with pre-programmed VR film. Fully immersive experience. Control: Education by routine oral information on the procedure from the treating cardiologist and patients received an informative flyer on the upcoming procedure	Pre-surgical Anxiety Questionnaire: STAI Second time point was within 1 week before the cardiac procedure
Rousseaux et al., 2022 [31]	37 (15/22)	64.7±13.4/63.3±11.5	CABG, MVR, AVR	Intervention: In addition to the daily standard care, a 20-minute virtual reality session wearing an HMD with goggles. Audiovisual display pre and post-operative Control: daily standard care pre and post-operative	Pre and post-operative Anxiety and Pain Method: Visual Analogue Scale
Keshvari et al., 2021 [34]	80 (40/40)	50.95±4.12/52.08±4.00	Coronary Artery Angiography	Intervention: A VR video headset with the ability to change 360-degree angle of view was used while playing VR video. A headphone was also used to play the distraction music. It was a 5-minute VR session. Control: no intervention or placebo was used	Pre-procedural Anxiety Questionnaire: Short form STAI Second time point was at the end of education and before the operative procedure
Morgan et al., 2021 [33]	64 (33/31)	68.7 ±11.5	Cardiac Catheterization	Intervention: 10-minute VR immersive video on a dedicated VR headset describing pre-procedural and procedural experience for the day of cardiac catheterization. Concurrent audio was provided through earplugs to complete the patient’s immersive experience Control: Information was provided by BHF information booklets, verbal explanation of the procedure by the pre-procedural assessment nurse, and BHF cardiac catheterization video	Pre and post-cardiac procedure Anxiety Questionnaire: Short form STAI
Laghlam et al., 2021 [20]	180 (90/90)	68.0[60.0-74.8]*	Post Cardiac Surgery drain removal	Intervention: VRx helmet 90-degree field of view with head tracking and patients could choose from five different immersive environments. VR session started at least 5 min before the drain removal and continued for 10 min after. Control: Kalinox, an equimolar mixture of oxygen and nitrous oxide was started 1 min before the drain removal and delivered continuously until 1 min after removal. Patients were warned orally before drains removal.	Pre and post-procedure Anxiety and Pain Method: NRS Outcome assessed before and immediately after the drain removal
Chang et al., 2021 [19]	33 (11/22)	Not mentioned	Atrial Fibrillation Catheter Ablation	Intervention: 3-minute pre-cardiac procedure education via HMD VR was given. Control: Paper-based written educational material	Peri-procedural Anxiety and Pain Method: Operator assessed the outcome during the procedure
Hendricks et al., 2020 [35]	20 (10/10)	69.5±6.9/63.4±9.1	CABG	Intervention: patients wore a headset for immersive VR experience and played a non-violent VR game in which the patients move their head and visual gaze to target objects in an energetic cartoon world. Control: non-VR tablet based game application with audiovisual-tactile stimulation as well as a defined objective was used.	Pre-operative Anxiety Assessment Questionnaire: STAI Pre and post intervention (20 minutes of using the tablet or control), questionnaires were administered.
Pouryousef et al., 2020 [32]	60 (30/30)	49.96±8.10/51.36±8.11	Coronary Artery Angiography	Intervention: the intervention consisted of showing calming images using a VR camera for 5 minutes. Control: received routine care before angiography	Pre-procedural Anxiety Questionnaire: STAI Questionnaire was given before the intervention and half an hour after the intervention
Bruno et al., 2020 [37]	32 (16/16)	83 (78.25-87)*	TAVI	Intervention: VR 3D glasses were worn during the TAVI procedure and patients could choose from 5 different calming videos. Control: No 3-D glasses used	Peri-procedural Anxiety and Pain Method: VAS Questionnaire was filled 1 day before and 1 day after TAVI

Intervention Characteristics

The intervention characteristics are provided in Table 2. Across all studies, fully immersive VR technology was utilized. The duration of the VR intervention varied between 5 and 20 minutes across different studies. In six studies, participants were exposed to distraction via exposure to calming videos [20,31,32,34,37] or playing a non-violent video game [35] through VR. The remaining four studies used VR technology to deliver cardiac procedure-related education [19,21,33,36].

Risk of Bias Assessment

**Figure 2:**
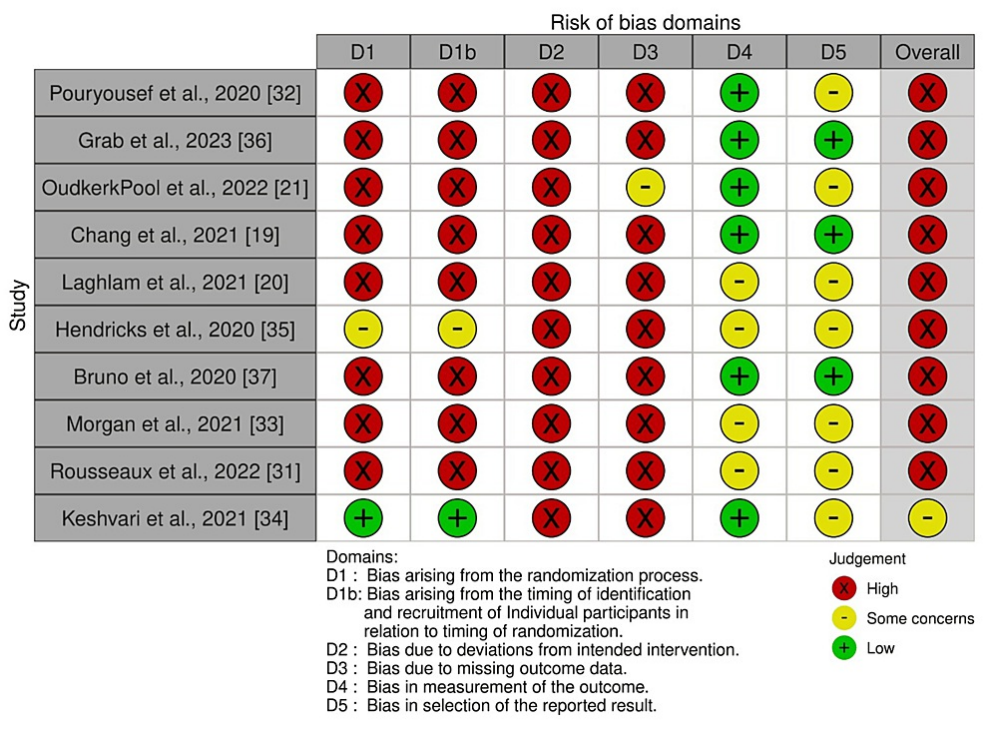
Risk of bias assessment of the randomized controlled trials using RoB 2 tool RoB: Risk of bias

The risk of bias in included studies is given in Figure 2. The randomization process was found to have a high risk of bias in all studies, except for Hendrick et al. [35], which had some concerns, and Keshvari et al. [34], which was assessed as low risk. Additionally, all studies were identified as having a high risk of bias due to deviations from the intended treatment and missing outcome data, except for Pool et al. [21], which had some concerns regarding missing outcome data. Regarding the measurement of outcome data, four studies were assessed with some concerns [20,31,33,35], while the remaining studies were identified as having a low risk. In terms of the selection of reported results, three studies had a low risk of bias [19,36,37], while the rest were assessed as having some concerns. Overall, the risk of bias for all included studies was determined to be high.

Outcome assessment

Anxiety Outcome

**Figure 3:**
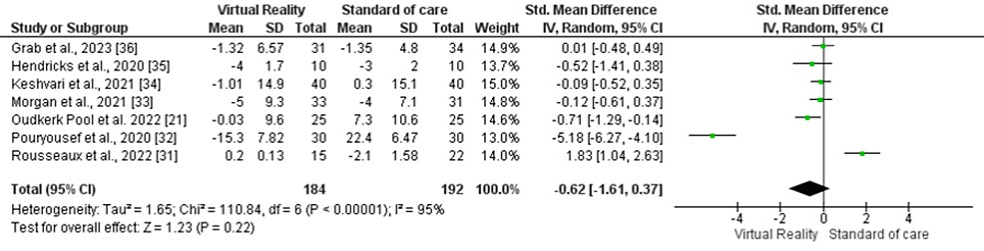
Forest plot for anxiety outcome

Seven studies were part of this comparison, involving a total of 376 participants (intervention arm: 184, control arm: 192). A random effects model was used for pooled assessment, revealing a statistically non-significant improvement in the VR arm (SMD -0.62, 95% CI -1.61, 0.37, p=0.22, I2=95%) compared to the control arm (Figure 3).

Subgroup Analysis

**Figure 4:**
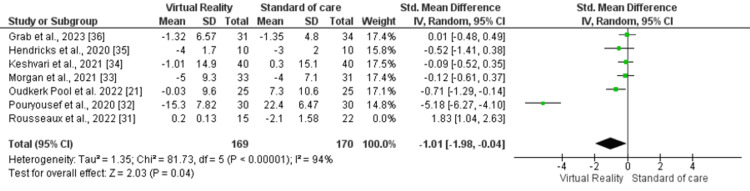
Forest plot of sensitivity analysis for anxiety outcome by taking out Rousseaux et al. study

Sensitivity analysis was performed by excluding the study by Rousseaux et al. [31] as it utilized a different anxiety assessment questionnaire, while the remaining six studies used the State-Trait Anxiety Inventory (STAI) questionnaire. In this analysis, a statistically significant improvement was observed in the VR arm (SMD -1.01, 95% CI -1.98, -0.04, p=0.04, I2=94%) compared to the control arm (Figure 4).

**Figure 5:**
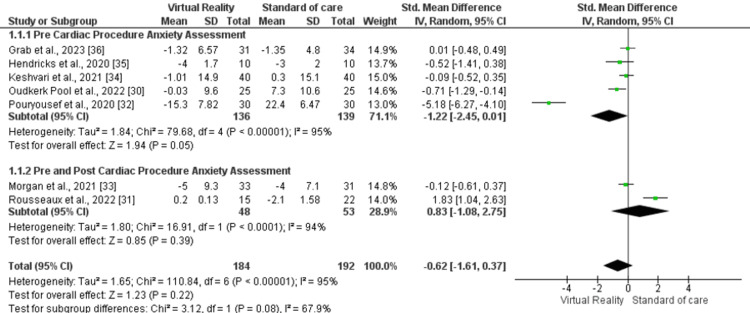
Forest lot of subgroup analysis for anxiety outcome based on timing of anxiety assessment

A subgroup analysis was conducted, stratifying the studies into two subgroups: those assessing anxiety before the cardiac procedure (SMD -1.22, 95% CI -2.45, 0.01, p=0.05, I2=95%) and those assessing anxiety after the cardiac procedure (SMD 0.83, 95% CI -1.08, 2.75, p=0.39, I2=94%). In both subgroups, no statistically significant difference was found between the VR and control arms (Figure 5). No significant difference between the two subgroups was identified as well (p=0.08, I2=67.9%).

**Figure 6:**
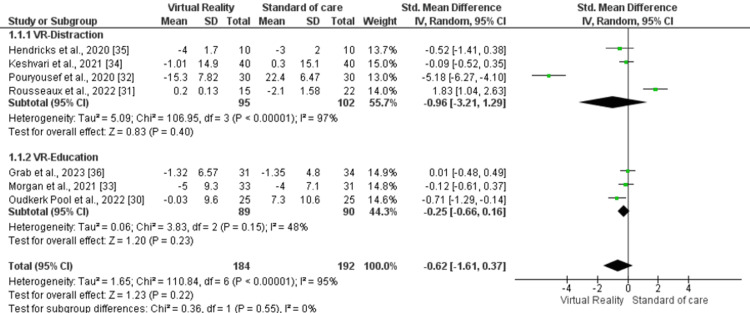
Forest plot of subgroup analysis for anxiety outcome based on VR utilization for distraction and cardiac-procedure related education VR: Virtual reality

In addition, another subgroup analysis was conducted based on the utilization of VR for distraction (SMD -0.96, 95% CI -3.21, 1.29, p=0.40, I2=97%) and providing cardiac procedure-related education (SMD -0.25, 95% CI -0.66, 0.16, p=0.23, I2=48%). In both subgroups, no statistically significant difference was found between the VR and control arms (Figure 6). The subgroup analysis did not reveal a significant difference between the two subgroups as well (p=0.55, I2=0%).

Assessment of Publication Bias

No significant publication bias was detected in the anxiety outcome, as indicated by Egger's test (p = 0.094).

Pain Outcome

Four studies, involving a total of 282 participants (intervention: 132, control: 150), assessed pain outcomes using different questionnaires and methodologies for measurement and reporting. Bruno et al. [37] (median 4 [IQR 3-4.8] vs (median 4 [IQR 2-6]) and Rousseaux et al. [31] reported no significant difference in pain improvement between the intervention and control groups. In contrast, Chang et al. [19] reported a significant improvement in peri-procedural pain in the VR group.

Laghlam et al. [20], however, reported significantly worse pain in the intervention group compared to the control group (median 5.0 [3.0-7.0] vs. median 3.0 [2.0-6.0]) immediately after the procedure. However, no difference in the improvement of pain was observed between the two arms after 10 minutes of the procedure.

Discussion

This review examined the effectiveness of VR- technology in improving procedure-related anxiety and pain compared to the standard of care in patients undergoing any type of cardiac procedure. No significant difference was found between the two groups in reducing cardiac procedure-related anxiety likely due to significant heterogeneity between the studies. However, after controlling for only the anxiety assessment tool, the VR group had significant improvement in anxiety compared with the control group among studies that utilized validated anxiety assessment STAI questionnaire. In addition, Rousseaux et al. [31] who assessed anxiety using the Visual Analog Scale (VAS), a validated psychological assessment tool that correlates well with STAI [38,39], confounded the results towards null which likely shows that, in addition to variability in the assessment tool, there were factors like study design, the nature of cardiac procedures, the assessment and reporting of results, and the methodologies for employing the intervention that may have contributed to the lack of significance. Among other studies, while most used a short STAI questionnaire, Pouryousef et al. [32] utilized the comprehensive Spielberg questionnaire. This form of STAI questionnaire assesses anxiety more comprehensively as the tool contains more items. It has excellent psychometric properties, good reliability, and is sensitive to treatment effects [40-42]. This can potentially explain the significantly improved effect estimate in the VR arm of this study, which further suggests the need for using validated anxiety assessment tools that can assess anxiety more comprehensively to identify the true potential of VR technology in mitigating cardiac procedure-related anxiety.

Some studies assessed pre- or post-procedural anxiety outcomes based on subjective interpretation. Chang et al. [19] compared paper with VR-based materials for atrial fibrillation catheter ablation, and peri-procedural anxiety was assessed by operators during the procedure who were blind to the two groups. It was noted that pre-procedural knowledge was better in the VR group as well as procedure-related anxiety was less in the intervention group compared to the control group. In addition, Bruno et al. [37] study, which utilized VAS to measure anxiety before and after the trans-catheter aortic valve implantation also found that VR intervention was associated with a significant anxiety reduction. However, Langham et al. [20] compared VR and an inhaled equimolar mixture of N2O and O2 (Kalinox®) for anxiety management during the removal of chest drains after cardiac surgery, and monitored analgesia/nociception index during the procedure for objective assessment of anxiety. The study also analyzed subjective self-reported anxiety using the numerical rating scale (NRS). It did not find any significant difference in procedure-related anxiety reduction between the intervention and the control groups using NRS. Interestingly, in both these studies, subjects in the intervention arm watched calming videos as a distraction during the procedure while the subjects received educational information on the cardiac procedure through the VR interface in the study conducted by Chang et al. [19].

The lack of standardization in tools used to measure anxiety is an important contributor to significant heterogeneity in results. These tools have ranged from STAI in the majority of studies to VAS, and even operator-reported anxiety measurement. In addition, there is notable variability in the clinical settings in which these studies have been conducted, ranging from coronary artery bypass graft surgery [31,35,36], patent foramen ovale repair [21], and aortic valve implantation [37] to atrial fibrillation catheter ablation, [19] post-cardiac surgery drain removal [20] and coronary angiogram+/- percutaneous coronary intervention [32-34]. While all interventions can provoke anxiety to some extent, anxiety is significantly more common among patients scheduled for high-risk procedures (like cardiac bypass or valve replacement procedures) compared to relatively low-risk procedures (like coronary angiography) [43]. This is often due to patients’ concerns regarding intra-procedural complications and post-operative recovery. Finally, there are gender differences in anxiety measurements peri-procedurally, as women tend to have higher STAI scores and hence, greater anxiety compared to men [44,45]. Some studies in our meta-analysis did not report gender distribution, while others demonstrated notable variation in the male-to-female ratio.

In this review, there was no significant improvement in pain in the VR group compared with the control group. This is at odds with the results of a recent meta-analysis, where VR was found to be effective in reducing pain in various painful situations. However, the strength of the findings was limited by significant clinical and statistical heterogeneity [46]. Bruno et al. [37] and Rousseaux et al. [31] did not demonstrate any significant improvement in pain using VAS while a demonstrable benefit was noted with VR in anxiety alleviation. Only Chang et al. [19] demonstrated improvement in pain with VR, and in this study, pain was assessed by the operator during the cardiac procedure. Laghlam et al. [20] interestingly, showed that pain in the intervention group was worse compared to the control group in the immediate postoperative period, but not after 10 mins of the procedure. One possibility for the variance found is the dosage of VR intervention, which seemed to remarkably differ in these studies. Another potential cause may be related to the variation in the type of procedure subjects underwent, VR equipment, and the VR environment. The utilization of VR for distraction or providing procedure-related education appeared to be equivocal for both anxiety and pain outcomes, primarily due to heterogeneity in the methodology and study design.

Limitations

Our systematic review and meta-analysis have several limitations that should be considered when interpreting the findings. First, there was notable heterogeneity among the included studies in terms of the VR technology employed. Different types of VR gadgets, software, and durations of application were utilized in the intervention arm, which may have influenced the results. Additionally, the comparator groups varied across studies, as different hospitals had their own standard of care protocols. Second, the timing of anxiety and pain assessments differed among the studies, with some assessing these outcomes before the cardiac procedure and others assessing them peri-procedurally. However, we conducted subgroup analyses to address this discrepancy. Third, the use of different questionnaires to measure anxiety and pain outcomes posed a limitation. To address this, we performed sensitivity analyses for anxiety outcomes, focusing on studies that utilized the same anxiety assessment tool.

## Conclusions

To the best of our knowledge, this is the first comprehensive systematic review and meta-analysis comparing VR technology with the standard of care for managing cardiac procedure-related anxiety and pain. Our findings revealed no significant improvement in anxiety between the intervention and control groups, likely due to significant heterogeneity between the studies. However, a significant anxiety reduction was noted in the VR group when accounting for the anxiety assessment tool, suggesting that VR technology may have the potential to enhance the patient experience by providing more comprehensive cardiac procedure-related education and acting as an effective distraction tool. Nevertheless, additional studies are warranted, utilizing standardized utilization of VR technology and consistent methods for assessing and reporting anxiety and pain. These future investigations will help elucidate the true potential of VR technology in improving the patient experience during cardiac procedures.
